# STATE OF HEALTH AND FUNCTIONAL AGING IN THE IMMIGRANTS AND MIGRANTS IN UKRAINE

**DOI:** 10.1093/geroni/igae098.3158

**Published:** 2024-12-31

**Authors:** Olena Tomarevska, Oleksandr Poliakov

**Affiliations:** D. F. Chebotarev Institute of Gerontology of the National Academy of Medical Sciences of Ukraine, Kyiv, Kyyiv, Ukraine; D. F. Chebotarev Institute of Gerontology of the National Academy of Medical Sciences of Ukraine, Kyiv, Kyyiv, Ukraine

## Abstract

The study of migration factors and the likelihood an impact on aging and health of respondents is important for the healthcare. This study is aimed to evaluate differences in the health of immigrants, migrants, and non-migrants among the 31-105 years. Online study will be perspectives to assess the health state and effectiveness of rehabilitation in migrant’s groups. Analysis of functional aging and health, sensory, cognition and physical capabilities of migrants and non-migrants in 31-105 years (1995-2023) offline and online study in Ukraine. For participants after 90, the impact of immigration processes of interstate and internal migration on health was determined. In persons 60-89 years old was relationship between functional aging and interstate immigration to Ukraine. Primary health assessment was analyzed the impact of outward migration and comparison with people who remained inside the country in Ukraine. In the results the trend are accelerator characters of functional aging. The leading indicators in monitoring health status and functional aging processes were the ability in centenarians to sit-to-stand up test (r=0.176, p< 0.05), in the older adults (r=-0.210, p< 0.05), in the online study (P=0.006) as well as the ability to remember three words after over time (r=0.174, p< 0.05) in ninety-year-olds. We found changes in these parameters, characterized by better indicators for migrants who emigrated and immigrated to the territory of Ukraine. This fact shows that people migrate with better health indicators, and these relationships health status at older persons and centenarian require monitoring and for rehabilitation programs.

